# Editorial: Integrating advanced high-throughput technologies to improve plant resilience to environmental challenges

**DOI:** 10.3389/fpls.2023.1218691

**Published:** 2023-05-31

**Authors:** Freddy Mora-Poblete, Parviz Heidari, Sigfredo Fuentes

**Affiliations:** ^1^ Laboratory of Forest Genetics and Biotechnology, Institute of Biological Sciences, University of Talca, Talca, Chile; ^2^ Faculty of Agriculture, Shahrood University of Technology, Shahrood, Iran; ^3^ Digital Agriculture, Food and Wine Sciences Group, School of Agriculture, Food and Ecosystem Sciences, Faculty of Science, The University of Melbourne, Melbourne, VIC, Australia; ^4^ Tecnologico de Monterrey, Escuela de Ingeniería y Ciencias, Monterrey, Mexico

**Keywords:** abiotic stress resilience, high-throughput technologies, data integration, multilevel biological data, bioinformatics resources, multi-omics

High-throughput technologies enable extensive omics datasets for genomics, transcriptomics, proteomics, phenomics, and metabolomics analysis. These advancements, accompanied by evolving bioinformatics tools, integrate omics-related data, providing critical information about plant molecular systems and their functions (Choi, 2019). These technologies significantly advance omics research in plants, investigating gene function, regulation, and adaptation. Furthermore, they contribute to the recovery of substantial plant diversity, vital for genetic improvement, food security, and conservation efforts (Kumar et al., 2021). By integrating multi-level biological data from genomics, transcriptomics, proteomics, and metabolomics, comprehensive investigations and insights into molecular aspects governing responses to abiotic stresses can be achieved.

This Research Topic integrates advanced high-throughput technologies, multi-omics, bioinformatics, systems biology, and artificial intelligence to explore plant stress and tolerance to environmental constraints. It includes nine original research articles, enhancing plant resilience to stressors like drought, cold, ultraviolet radiation, flooding, and low-nitrogen stress. The articles encompass important plant species: rice, potato, cabbage, sugarcane, poplar, Antarctic moss (*Pohlia nutans* and *Leptobryum pyriforme*), and an endangered plant species, *Myricaria laxiflora*. Additionally, a review examines recent progress in genome engineering and the role of CRISPR-Cas9-mediated genome editing in sustainable agriculture.

Various cutting-edge technologies are explored in this Research Topic to enhance plant resilience against environmental challenges. These include transcriptomics, proteomics, metabolomics, and phenomics. Dwivedi et al. conducted the first study to employ high-throughput phenomics parameters for selecting reproductive stage drought stress (RSDS) tolerance in near-isogenic lines (NILs) of rice. Four traits were used: projected shoot area, water use, transpiration rate, and red-green-blue and near-infrared values. Results showed the potential role of quantitative trait loci (QTLs) in improving water use efficiency (WUE) and identified eleven NILs based on phenomics traits and performance under imposed drought in the field. In conclusion, high-throughput phenomics-based phenotyping enables efficient and rapid assessment of plant traits, facilitating the selection of stress-tolerant genotypes in challenging environments.

The integration of transcriptomics and proteomics is of paramount importance in advancing our understanding of complex biological systems. In this regard, Li et al. investigated the regulatory mechanisms of flooding stress by integrating transcriptomic and proteomic analyses in roots of *Myricaria laxiflora* (an endangered plant) during nine-time points under flooding and post-flooding recovery treatments. Genes related to auxin, cell wall, calcium signaling, and MAP kinase signaling were greatly down-regulated exclusively at the transcriptomic level during the early stages of flooding. Six groups of differentially expressed proteins were exclusively identified at the proteome level, showing specific expression patterns. These proteins were associated with glycolysis, major carbohydrate metabolism, redox reactions, and G-protein signaling. They played important roles in energy generation and redox homeostasis during flooding stress. Post-flooding recovery showed activation of genes related to ROS scavenging, mitochondrial metabolism, and development. In addition, the genes/proteins related to redox, hormones, and transcriptional factors also played vital roles in flooding stress of *M. laxiflora*. These findings enhance our understanding of flooding stress mechanisms and aid in preserving endangered plants in flood-prone areas. Chen et al. performed an integrated metabolomics and transcriptomics analysis to examine changes in metabolites and regulatory pathways in *Populus tomentosa* under low-N stress. Nitrogen is essential for plant growth and development, but little is known about how trees regulate their metabolism under N deficiency. RNA sequencing revealed differentially expressed genes related to carbohydrate metabolism and hormone pathways. Integrated metabolomics and transcriptomics analyses revealed a co-expression pattern of metabolites and genes. These findings provide insights into the metabolic and molecular mechanisms underlying N and carbon interactions in poplar.

Ponce et al. and Sajad et al. conducted transcriptomic studies to explore the genetic and molecular mechanisms underlying plant responses to different stress conditions such as drought and cold. Ponce et al. used RNA-sequencing study to investigate the drought response of two Andigenum varieties of potato with different drought tolerance. Comparative transcriptome analysis revealed significant early differences in the responses of the tolerant variety, suggesting the importance of rapid response. The tolerant variety exhibited more effective ABA synthesis and mobilization, feedback regulation, and differential expression of genes involved in cell wall reinforcement and remodeling. These findings provide insights into molecular bases of drought tolerance mechanisms and pave the way for improving potato resistance to drought stress. Sajad et al. conducted a genome-wide study on cabbage (*Brassica oleracea* var. capitata L.) and highlighted the crucial roles of the Hsp90 gene family in growth and development under cold conditions. This study discovered 12 BoHsp90 genes classified into five groups. Promoter evaluation revealed stress-related and hormone-responsive cis-elements in the genes. RNA-seq data analysis showed tissue-specific expression of BoHsp90-9 and BoHsp90-5, with six genes induced by cold stress, indicating their important role in cold acclimation and overall growth and development.

Integrative transcriptomics and metabolomics data analysis involves combining and analyzing data from transcriptomic studies (gene expression) and metabolomic studies (metabolite profiling) to gain a comprehensive understanding of biological processes. In this context, two studies investigated the molecular mechanisms underlying the adaptation of Antarctic moss species, *Pohlia nutans* and *Leptobryum pyriforme*, to UV-B radiation. Liu et al. studied the molecular mechanism of this adaptation and used transcriptomics and metabolomics to profile *Leptobryum pyriforme* moss under UV-B radiation. The results showed differentially expressed genes involved in UV-B signaling, flavonoid biosynthesis, ROS scavenging, and DNA repair. Flavonoids were the most changed metabolites, and the UVR8-mediated signaling, jasmonate signaling, flavonoid biosynthesis pathway, and DNA repair system contributed to adaptation. On the other hand, Liu et al. assembled the high-quality genome sequence of the Antarctic moss (*Pohlia nutans*) with 698.20 Mb and 22 chromosomes, revealing genomic features that aid in its adaptation to the extreme environment. The large size of the genome is due to a high proportion of repeat sequences and a recent whole-genome duplication event. The genome also shows evidence of massive gene duplications and expansions of gene families that facilitate neofunctionalization. The moss exhibits expanded gene families involved in phenylpropanoid biosynthesis, unsaturated fatty acid biosynthesis, and plant hormone signal transduction, likely supporting its Antarctic lifestyle. Additionally, the moss has developed adaptive strategies for UV-B radiation through the expansion and upregulation of genes encoding DNA photolyase, antioxidant enzymes, and flavonoid biosynthesis enzymes. The findings of these investigations provide insights into the adaptation of Antarctic moss to polar environments and assessing global climate change’s impact on Antarctic land plants.

Malviya et al. and Xu et al. utilized advanced high-throughput technologies with the aim of advancing research on the breeding improvement of sugarcane. In the study by Malviya et al., advanced high-throughput sequencing techniques were likely employed to explore the diversity of root-associated microbiomes in sugarcane species. Results revealed a significant rhizospheric diversity across progenitors and close relatives. The rhizosphere microbial abundance of modern sugarcane progenitors was at the lower end of the spectrum, indicating the potential of introgression breeding to improve nutrient use and disease and stress tolerance of commercial sugarcane. On the other hand, Xu et al. examined the effects of Indole-3-butyric acid (IBA) on sugarcane seedling growth and gene expression during root development. Results showed significant growth promotion and accumulation of plant hormones at 100 ppm IBA. Transcriptomic analysis revealed significant differentially expressed genes, mainly involved in metabolic, cellular, and single-organism processes. The study also analyzed the expression of genes related to plant hormones and signaling pathways. The findings provide new insights into the IBA response to sugarcane bud sprouting, which could aid in the breeding improvement of sugarcane.

Finally, one review article was included in this Research Topic. Ali et al. reviewed the recent progress in genome engineering and the role of CRISPR-Cas9-mediated genome editing in sustainable agriculture. Genome editing technology, particularly CRISPR-Cas9-based systems, offers significant potential for precise trait targeting, including enhancing resistance to microorganisms. Non-genetically modified plants can be developed using base editing systems. Disease-resistant crops have been produced using gene editing and are likely to gain greater public acceptance than conventionally genetically modified plants. Genome editing can enhance crop productivity to meet current and future nutritional requirements worldwide.

In summary, the Research Topic brought together recent findings and literature on utilizing advanced high-throughput technologies to enhance plant resilience against environmental challenges. These insights have shed light on how plants can withstand and adapt to environmental stress by applying such technologies (Veley et al., 2017; Gogolev et al., 2021).

## Author contributions

FM-P wrote the first draft of the manuscript. All authors contributed to the conception of the Research Topic, manuscript revision, editing, and approved the submitted version.

## References

[B1] Choi H. K. (2019). Translational genomics and multi-omics integrated approaches as a useful strategy for crop breeding. Genes Genomics 41, 133–146. doi: 10.1007/s13258-018-0751-8 30353370 PMC6394800

[B2] Gogolev Y. V. Ahmar S. Akpinar B. A. Budak H. Kiryushkin A. S. Gorshkov V. Y.. (2021). Omics, epigenetics, and genome editing techniques for food and nutritional security. Plants 10 (7), 1423. doi: 10.3390/plants10071423 34371624 PMC8309286

[B3] Kumar A. Anju T. Kumar S. Chhapekar S. S. Sreedharan S. Singh S.. (2021). Integrating omics and gene editing tools for rapid improvement of traditional food plants for diversified and sustainable food security. Int. J. Mol. Sci. 22 (15), 8093. doi: 10.3390/ijms22158093 34360856 PMC8348985

[B4] Veley K. M. Berry J. C. Fentress S. J. Schachtman D. P. Baxter I. Bart R. (2017). High-throughput profiling and analysis of plant responses over time to abiotic stress. Plant Direct 1 (4), e00023. doi: 10.1002/pld3.23 31245669 PMC6508565

